# Zone of Polarizing Activity Regulatory Sequence Mutations/Duplications with Preaxial Polydactyly and Longitudinal Preaxial Ray Deficiency in the Phenotype: A Review of Human Cases, Animal Models, and Insights Regarding the Pathogenesis

**DOI:** 10.1155/2018/1573871

**Published:** 2018-02-13

**Authors:** Mohammad M. Al-Qattan

**Affiliations:** King Saud University, Riyadh, Saudi Arabia

## Abstract

Clinicians and scientists interested in developmental biology have viewed preaxial polydactyly (PPD) and longitudinal preaxial ray deficiency (LPAD) as two different entities. Point mutations and duplications in the zone of polarizing activity regulatory sequence (ZRS) are associated with anterior ectopic expression of Sonic Hedgehog (SHH) in the limb bud and usually result in a PPD phenotype. However, some of these mutations/duplications also have LPAD in the phenotype. This unusual PPD-LPAD association in ZRS mutations/duplications has not been specifically reviewed in the literature. The author reviews this unusual entity and gives insights regarding its pathogenesis.

## 1. Introduction

**Figure 1:**
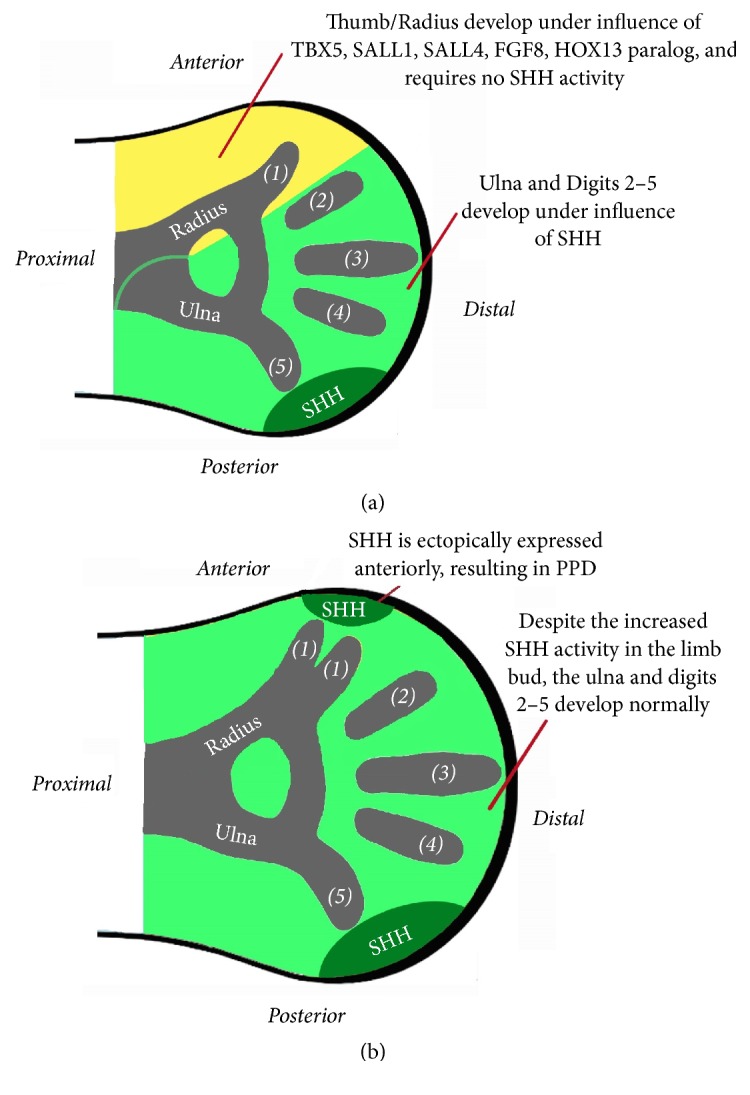
(a) SHH is posteriorly located in the Zone of Polarizing Activity (ZPA, marked with dark green). The SHH protein is a diffusible morphogen. Normally, SHH activity (light green color) extends anteriorly to reach digit 2. There is no SHH expression or activity in the zones of digit 1/radius. (b) Anterior ectopic expression of SHH results in PPD (preaxial polydactyly).

Preaxial polydactyly (PPD) indicates the presence of extra digit(s) on the preaxial side of the hand or foot [1]. Genetically, PPD is classified according to either the Temtamy-McKusick [2] or the Winter-Tickle [3] classification. The former classifies PPD into 4 types: PPD I is duplication of a biphalangeal thumb, PPD II is isolated triphalangeal thumb or thumb duplication with a triphalangeal component, PPD III is polydactyly of the index finger, and PPD IV is polysyndactyly of the thumb. The latter classification includes syndromes in which PPD is a constant feature such as the triphalangeal thumb-polysyndactyly syndrome (TPTPS, MIM 188770), Werner syndrome (Tibial Hypoplasia, Polysyndactyly Triphalangeal Thumb Syndrome or THPTTS, OMIM 188770), and Laurin-Sandrow syndrome (mirror-image polydactyly of the hands and feet, absent tibiae, and duplication of the fibulae; OMIM 135750). In 2013, it was proposed that LPAD should be included in the PPD spectrum [4]. The normal development of the thumb/big toe requires the absence of Sonic Hedgehog (SHH) activity in the most anterior part of the mesoderm, which is the area of the developing digit 1. The normal expression/activity of SHH participates in the normal development of the ulna/fibula as well as digits 2–5 (Figure 1(a)). The abnormal anterior ectopic expression of SHH will result in PPD as shown in Figure 1(b) [5]. Furthermore, the degree of PPD will depend on the degree of abnormal ectopic SHH expression (duplication of a biphalangeal preaxial digit is seen with minor SHH ectopic expression while mirror-image PPD is seen with extreme SHH ectopic expression) [5–7].

Embryologically, the thumb/radius and big toe/tibia (also known as the preaxial rays) develop under the influence of ectodermal Fibroblast Growth Factor 8 (FGF8) and the expression of T-BOX5 (TBX5), HOX, SALL1, and SALL4 in the anterior mesoderm (Figure 1(a)). Hence, deficiency of FGF8/TBX5/SALL1&4 leads to longitudinal preaxial ray deficiency (LPAD); and this is considered as failure of formation of the preaxial ray along the anteroposterior axis [8]. The spectrum of LPAD starts with isolated mild hypoplasia of the thumb/big toe and ends with complete absence of the entire preaxial ray [9].

The zone of polarizing activity regulatory sequence (ZRS) is the main controller of SHH activity in the limb bud [13]. Normally, SHH expression is restricted posteriorly within the Zone of Polarizing Activity (ZPA) and it moderates the anteroposterior axis of limb development [6]. Although the SHH protein diffuses in an anterior direction, it does not normally reach the most anterior part of the mesoderm where the preaxial ray develops (Figure 1(a)). The ZRS is located in humans on chromosome 7 and in mice on chromosome 5, within intron 5 of* LMBR1* (about 1 Mb telomere of the* SHH* gene). There are several point mutations/duplications in the ZRS in humans and in mice models [14]. They are all associated with anterior ectopic expression of SHH and result in various forms of PPD. Some of these point mutations/duplications in humans and mice models will also have LPAD in the phenotype (Table 1). In the current review, this combined phenotype will be named “PPD-LPAD association.”

### 1.1. ZRS Point Mutations at Position 404 in Humans

Point mutations at position 404 appear to be a hotspot for PPD-LPAD associations. The phenotype is known as Werner syndrome. In this syndrome, tibial hypoplasia is usually more commonly seen than hypoplasia of the radius.

One family was found to have G>A transition at position 404 of the ZRS. All family members had PPD except the proband who also had bilateral tibial hemimelia and mild radius dysplasia [16]. Another family with 404 G>A ZRS mutation had PPD-five-fingered hand—tibial hemimelia phenotype [17].

The 404 G>T ZRS mutation was reported in one family and resulted in a variable phenotype including PPD in the hands and feet, opposable and nonopposable triphalangeal thumbs, broad big toes/first metatarsals (which may be considered as “Forme Fruste” PPD), cutaneous syndactyly, and concurrent preaxial and postaxial polydactyly in one case. The LPAD phenotype in the same family was also variable and included tibial hemimelia, slightly short radius, small radius and ulna, and hypoplastic radius with thumb hypoplasia. In this particular mutation, LPAD of the upper limbs was more commonly seen than LPAD of the lower limbs [18].

In contrast to the variable phenotype of the 404 G>T ZRS mutation, the 404 G>C ZRS mutation resulted in a more consistent phenotype. All affected patients had triphalangeal thumbs in the hands and PPD in the feet. Tibial defects and bowing of the fibula were a common feature. Interestingly, one patient had duplicated fibulae [16].

### 1.2. ZRS Point Mutations at Positions 406, 417, and 619 in Humans

The 406 A>G ZRS mutation was reported in two families with features of Werner mesomelic syndrome (triphalangeal thumbs, PPD, and tibial defects) [19].

The ZRS 417 A>G mutation presented with a very unique phenotype of mirror-image polydactyly of the four extremities and bilateral tibial deficiency [20]. Interestingly, the same mutation in a mosaic form resulted in isolated triphalangeal thumb or thumb duplication with a triphalangeal component (PPD type II) [20].

The ZRS 619 C>T mutation was also unique because all defects were confined to the upper limbs [21]. In the upper limbs, the PPD presented as triphalangeal thumb, polydactyly of the index finger, duplication of a biphalangeal thumb, and rudimentary PPD (i.e., a preaxial nubbin). The LPAD was represented by thumb aplasia in two family members. More interesting, a third family member had bilaterally absent thumb and complete aplasia of the radius on the right and partial aplasia of the radius on the left. The same patient had absence of the left kidney and cardiac defects. This phenotype is a VACTERL phenotype (Vertebral defects, imperforate Anus, Cardiac defects, TracheoEsophageal fistula, Renal defects, and preaxial upper Limb defects) since the patient had three out of the six VACTERL diagnostic criteria. The pathogenesis of this phenotype will be discussed in more detail later in the review.

### 1.3. ZRS Point Mutations at Position 402 in Humans

Previous authors have emphasized that ZRS point mutations resulting in Werner syndrome (which has PPD-LPAD association in the phenotype) have “strong” ectopic anterior SHH expression [16]. Further evidence regarding this strong anterior expression comes from appearance of mirror-image duplication of the hands and feet in some patients with ZRS point mutations [20]. Experimentally, transplantation of few SHH expressing cells to the anterior limb bud results in PPD. As more cells are transplanted, higher degrees of PPD are seen and eventually mirror-image polydactyly is observed [6]. Perhaps the most convincing evidence that the PPD-LPAD association requires a strong SHH anterior expression comes from observations of the phenotypes of ZRS 402 C>T mutations in the hetero- and homozygous forms. In the heterozygous form, the mutation results in isolated PPD; and, in the homozygous form, the mutation results in a Werner syndrome phenotype [22].

### 1.4. Duplications Encompassing the ZRS in Humans

Most duplications encompassing the ZRS in humans result in either triphalangeal thumb-polysyndactyly syndrome (TPTPS, MIM 174500) or Haas-type polysyndactyly (also known as syndactyly type IV, MIM 186200) [15, 16, 23].

Lohan et al. [15] reported on three unrelated families with overlapping microduplications (<80 Kb) encompassing the ZRS resulting in Laurin-Sandrow syndrome phenotype. The fact that mirror-image duplications were seen with ZRS duplications indicated that some duplications resulted in strong SHH enhancements. Indeed, Wu et al. [24] reported on a Chinese family with a ZRS duplication resulting in complete syndactyly of all digits with polydactyly (syndactyly type IV, OMIM 186200) and concurrent tibial hypoplasia [24].

Our review shows that all cases with ZRS duplications show syndactyly in the phenotype; and with strong SHH enhancements PPD-LPAD association may also be seen.

### 1.5. Mice Models Involving the ZRS and Showing PPD-LPAD Association in the Phenotype

The Sasquatch (Ssq) is a mouse mutation within the ZRS and it arose through a transgenic insertion [13, 25]. Heterozygotes have isolated PPD on the hind feet only. Homozygotes have higher grades of PPD in both the forelimbs and hind limbs; and some mice also had shortening of the long bones. In the homozygous phenotype, the reduction of long bones was more prominent in the hind limbs and usually involved both the tibia and fibula.

The hemimelic extra toe (Hx) mouse polydactylous mutation was also mapped to the ZRS [26]. Mutants have PPD and hypoplasia of the tibia/radius. This mouse model seems to be a better corollary to the accounts reported in humans than the Ssq model because the reduction of long bones is focused on the preaxial bones only.

### 1.6. Why Do Mutations/Duplications of the ZRS only Affect the Limb Bud?

Besides the limb bud, SHH is expressed in the brain, neural floor plate, and epithelial linings of the lung and gut. Yet, ZRS mutations/duplications have no neural or epithelial defects. The reason for that is the fact that ZRS is a limb-specific enhancer. Brain (SBE1–4) and floor plate (SFPE1, 2) enhancers are located within the* SHH* gene. Epithelial enhancers are located in next gene* (Rnf32)*. The* LMBR1* gene (where the ZRS is located) is in the next gene over [27].

### 1.7. The Normal Development of ZPA and SHH

**Figure 2:**
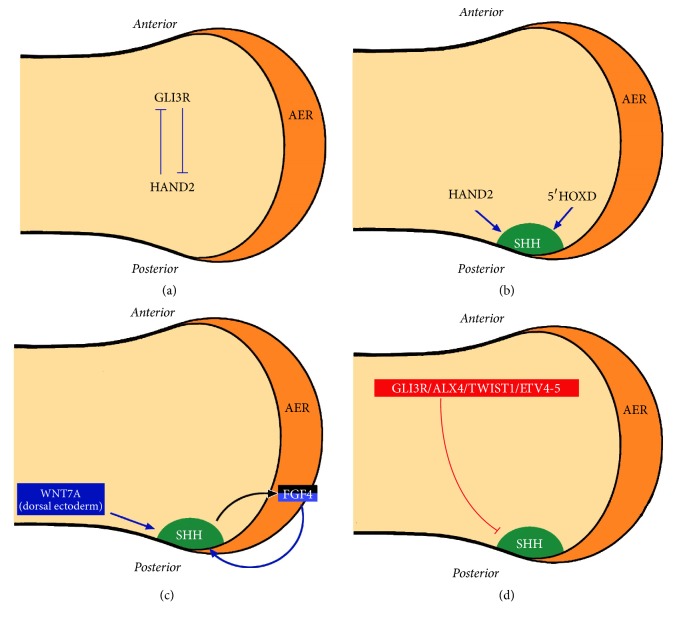
(a) Prepatterning establishes anteroposterior polarity of the early limb bud as a result of the antagonistic activity of GLI3R anteriorly and HAND2 posteriorly. This results in establishing competency for ZPA (Zone of Polarizing Activity) induction in the posterior mesoderm. (b) Next, SHH is induced within the ZPA (colored dark green) by HAND2 and 5′HOXD. (c) Following the expression of SHH, its maintenance is through WNT7A and the SHH-FGF4 feedback loop. (d) “Restriction” of SHH (preventing its transcriptional activation at the most anterior part of the mesoderm) is mediated by the “negative” regulators of SHH: GLI3R, ALX4, TWIST1, and EVT4/5. Note that other inhibitory regulatory modules exist such as the module described by Petit et al. [10].

The normal development of the ZPA (in which SHH is located) passes through 3 stages [28, 29] (Figure 2). “Initiation or prepatterning” (Figure 2(a)) establishes the normal anteroposterior polarity in the early limb bud by the normal expression of the ZPA posteriorly. This normal polarity occurs because of the antagonistic interaction between GLI3R (the repressor form of GLI3) and HAND2 in the early limb bud, resulting in the restriction of GLI3R anteriorly and HAND2 posteriorly [28].

In the “induction” stage (Figure 2(b)), the normal expression of SHH within the ZPA occurs under the influence of HAND2 and 5′HOXD. These inducers are located in the posterior mesoderm of the limb bud and are known as the “positive” regulators of SHH. Loss of function of these positive regulators in experimental animals will result in a reduction of SHH activity [Trachini et al., 2006].

Finally, “maintenance/restriction/control” of SHH activity will be described separately. SHH activity within the ZPA is “maintained” by FGF4 (expressed in the posterior part of the AER) and by WNT7A (expressed in the dorsal ectoderm). SHH induces FGF4 in the overlying AER and FGF4 will then help maintain the expression of SHH; and this is known as the SHH-FGF4 feedback loop [30, 31] (Figure 2(c)). “Restriction” of SHH (preventing its transcriptional activation at the most anterior part of the mesoderm) is mediated by the “negative” regulators of SHH: GLI3R, ALX4, TWIST1, and EVT4/5 (Figure 2(d)). This restriction of SHH expression “prevents” the formation of PPD (Figure 1(b)). Finally, the ZRS is the main “controller” of SHH activity in the limb bud as mentioned earlier in the Introduction [13]. However, other limb-specific cis-regulatory elements of SHH exist. Early evidence came from the fact that several families with PPD and linkage to 7q36 do not have any ZRS defects [32, 33]. More recently, Petit et al. [10] reported on a “PPD-hypertrichosis” phenotype in a French family. No pathogenic variants or copy number variations were found in the ZRS. Instead, there was loss of a 2026 bp region in the gene desert 240 kb from the* SHH* promotor in all affected patients. Transfection experiments identified an inhibitory cis-regulatory element that is believed to be active in the anterior margin silencing anterior expression of Shh.

### 1.8. How Are Mutations/Duplications/of the ZRS Translated into an Anterior Ectopic SHH Expression in the Limb Bud?

As mentioned earlier, the SHH protein does not normally reach the most anterior part of the limb bud. There are two different ways by which SHH can reach the most anterior part of the limb bud (which results in PPD): the deficiency of SHH negative regulators (GLI3, ALX4, TWIST1, and ETV4/5) and the expansion of the ZPA boundary itself.

Lettice et al. [27] showed that members of two groups of ETS transcription factors act directly at the ZRS: the normal binding of GABP *α*/ETS1 at multiple sites of the ZRS regulates the ZPA boundary, while the normal binding of ETV4/5 restricts SHH expansion outside the ZPA. With ZPA point mutations/duplications, changes in ETS binding will lead to anterior ectopic expression of SHH and PPD [27].

### 1.9. How Do the Transcription Factors ETV4/5 Restrict Anterior SHH Expansion?

ETV4/5 activation is dependent on the overlying fibroblast growth factors within the apical ectodermal ridge (AER). Furthermore, ETV4/5 are expressed in the distal mesoderm along the entire distal edge (just below the AER). There is sufficient genetic and biochemical evidence suggesting that the anterior restriction of SHH by ETV4/5 is through regulation of the dimerization of Twist1 and Hand2 [34]. Twist1 and Hand2 are members of the basic helix-loop-helix family. They antagonize each other by forming protein heterodimers. The interaction occurs at the E-box DNA sequence elements to form the heterodimer [35]. As mentioned earlier, Alx4 is a “negative” regulator of SHH activity. This means that it is expressed anteriorly in the mesoderm and it acts to restrict SHH anterior expansion. Mutations in mouse* Alx4* gene cause Strong's luxoid polydactyly and anterior ectopic expression of SHH [36]. Homozygotes show a very strong anterior SHH expression. As expected, homozygotes have mirror-image polydactyly (the highest grade of PPD) as well as reduction of the radius and tibia (i.e., PPD-LPAD association) [37].

In contrast, Hand2 is a “positive” regulator of SHH activity. This means that it is expressed posteriorly in the mesoderm and helps induce posterior SHH expression. Hence, Hand2-deficient mice lack SHH expression [38], while misexpression of Hand2 anteriorly will induce anterior ectopic SHH expression and mirror-image polydactyly [39, 40]. More interesting, Hand2 also binds to the ZRS [41].

### 1.10. What Are the Most Prominent Molecular Changes Associated with Anterior SHH Expression?

Several animal models with anterior SHH expression [36, 42–44] showed similar molecular changes in the limb bud. These changes include the abnormal anterior ectopic expression of Fgf4 in the AER and the abnormal anterior ectopic expression of 5′Hox in the mesoderm of the early limb bud.

Normally, the AER has a high posterior expression of FGF4 and this is partly mediated through the SHH-FGF4 feedback loop [30]. In contrast, FGF8 is normally highly expressed along the entire AER. Animal models with ectopic anterior expression of SHH in the mesoderm showed the abnormal anterior expression of FGF4 in the AER [36, 42–44].

The other major molecular change with anterior SHH expression is the abnormal HOX expression. In normal early development, HOXD are expressed in the autopod in a collinear way, with lower numbers (i.e., the 3′ group) expressed anteriorly and the 5′ group (i.e., HOXD 10–13) expressed posteriorly. This is known as the “Russian dolls” strategy of expression [45]. The second wave of HOX expression is known as the “reverse collinearity” pattern and it occurs under the influence of both SHH and the regulatory sequences centromeric to the HOXD cluster [45]. In this phase, HOXD 13 is transcribed all over the mesoderm (including the anterior thumb area); while HOXD 10–HOXD 12 are expressed in digits 2–5 only. With anterior ectopic expression of Shh, animal models show anterior ectopic expression of the Hoxd13 during early development [37]. This is expected since Hoxd13 is known to be induced by Shh [46].

A third molecular change with anterior SHH expression is in the pattern of SALL1 expression. Normally, SALL1 expression in the mesoderm is posteriorly biased. In chick spalt homologous, anterior Shh expression leads to an expanded csall expression anteriorly [47]. This is not surprising because other animal models showed that Shh signaling participates in the expression of Sall1 [11].

Finally, SHH-GLI3 interactions play an important role in preaxial ray development [48]. The normal posterior SHH expression maintains the GLI3 transcription factor as a full-length activator (GLI3A). Anteriorly (where there is no SHH expression), GLI3 is processed into a repressor (GLI3R). The gradient of GLI3R transduces SHH signaling and disturbances of the normal SHH-GLI3 pattern affect the regulation of digit number and identity [49].

### 1.11. The Pathogenesis of PPD-LPAD Association in ZRS Mutations/Duplications

The molecular interactions within the mesoderm as well as mesodermal-ectodermal interactions within the developing limb bud are too complex to simply state that there is a single pathogenesis explaining the PPD-LPAD association seen with strong anterior SHH expression.

**Figure 3:**
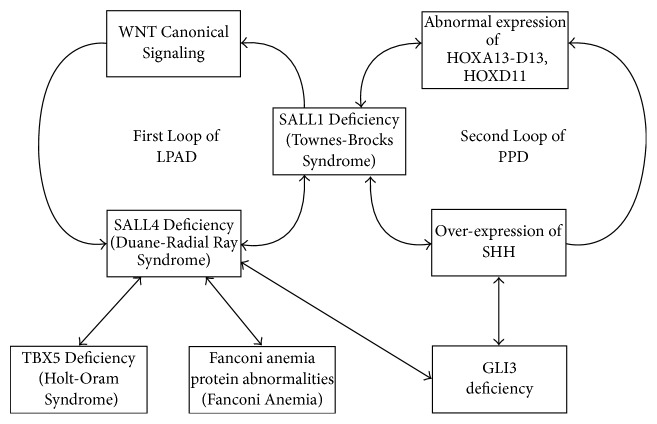
The interactions between the LPAD and PPD loops may explain the PPD-LPAD association related to ZRS mutations/duplications (see text for details). Note that both loops share SALL1 and GLI3. Deficiency of SALL1 (within the LPAD loop) will affect both the expression of SHH and the HOX system as described by Kawakami et al. [11]. Hence, the Townes-Brocks phenotype may present as LPAD only, PPD only, or PPD-LPAD association. GLI3 deficiency will result in anterior ectopic expression of SHH and PPD, since GLI3 is a negative regulator of SHH. Simultaneously, GLI3 deficiency may result in SALL4 deficiency as described by Akiyama et al. [12]. Hence, GLI3 is another important link between the two loops.

One possible pathogenesis is through the interactions of two mesodermal loops shown in Figure 3. The first loop is labelled as the LPAD loop because its components are known to result in syndromes in which LPAD is a feature. The key player of the first loop is SALL 4. SALL4 interacts with TBX5 to regulate the development of the thumb, radius, and heart [50]. There is also evidence that SALL4-Fanconi anemia complementation group L interactions exist [51]. Furthermore, SALL4-GLI3 system interactions exist and the system functions upstream of the Shh-expressing ZPA and Fgf8-expressing AER in both the forelimbs and hind limbs [12]. Finally, SALL4 forms a dimer with SALL1 and both cooperate in anorectal, heart, and kidney development [52]. SALL1 could then enhance the WNT canonical signaling [53] which could result in SALL4 activation via the interactions of LEF1 and TCF (both LEF1 AND TCF are WNT-related transcription factors) at the SALL4 promoter [54]. This loop explains the overlapping phenotypes of four syndromes: Duane-radial ray syndrome with SALL4 mutations (MIM 607323); Fanconi anemia (MIM 227650); Holt-Oram syndrome with TBX5 mutations (MIM142900); and Townes-Brocks syndrome with SALL1 mutations (MIM 107480). SALL1 of the LPAD loop becomes the key player of the second PPD loop. It is interesting to note that Townes-Brocks syndrome is the only LPAD syndrome in which PPD is commonly seen in the phenotype; and this is to be expected since SALL1 is the link between the two loops. SALL1 interacts with SHH and the HOX system (19]. Finally, the SHH anterior expression results in abnormal 5′HOX anterior expression in early development as mentioned earlier. The interactions between these two loops may explain the pathogenesis of PPD-LPAD association. These interactions are probably through SALL1 and GLI3 because both are shared by both loops (Figure 3).

A second possible pathogenesis of PPD-LPAD is through the overexpression of HOXD 11–HOXD 13 in the anterior mesoderm during early development which occurs secondary to anterior ectopic expression of SHH. In an animal model, Yang et al. [7] showed that the anterior ectopic expression of Shh results in an anterior ectopic expression of Hoxd 11–Hoxd 13. Zákány et al. [55] showed that the ectopic expression of Hoxd 11–Hoxd 13 in experimental animals was associated with Shh anterior ectopic expression and the phenotype varied among animals to include PPD only, mirror-image polydactyly with double ulnae, and LPAD (the latter was manifested by loss of the preaxial digit). This phenotypic variation is very similar to the various phenotypes of ZRS mutations/duplications, including the PPD-LPAD association. However, the ectopic expression of 5′HOX is upstream of SHH and, hence, it does not fully explain the downstream pathogenesis of PPD-LPAD association.

### 1.12. How Can We Explain the VACTERL Phenotype with ZRS Mutations?

Al-Qattan et al. [21] showed that ZRS 619 C>T mutation may result in a VACTERL phenotype (without PPD). Most VACTERL cases are sporadic and their genetic bases are unknown [56]. Animal models link the VACTERL phenotype to Shh signaling [57]. In humans, mutations and deletions of* FOXF1* (which is linked to SHH signaling) result in a VACTERL phenotype [58]. Furthermore,* HOXD13* mutations may result in a VACTERL phenotype; and, as mentioned earlier, HOXD13 is a downstream target of SHH [59]. All these cases as well as the case reported by Al-Qattan et al. [21] link the SHH signaling to at least some cases of the VACTERL phenotype. The VACTERL phenotype in ZRS mutations may also be explained by the interactions of SHH with the LPAD loop in Figure 1.* SHH* gene mutation analysis was previously reported to be negative in patients with VACTERL phenotype [60]. Our review suggests that screening for ZRS mutations and other genes related to SHH signaling (such as* FOXF1*,* HOXD 13*,* GLI 2*, and* GLI 3* genes) may be more appropriate than screening for* SHH* gene mutations in these patients.

## Figures and Tables

**Table 1:** Human ZRS mutations/duplications in which there is PPD-LPAD association in the phenotype.

ZRS mutations/duplications	The phenotype
*(A) Point mutations*	
ZRS 402 C>T	Werner syndrome phenotype
ZRS 404 G>A	Either Werner phenotype or PPD-five-fingered hand-tibial hemimelia phenotype
ZRS 404 G>T	Variable phenotypes of PPD-LPAD association
ZRS 404 G>C	Triphalangeal thumbs in the hands and PPD/tibial defects in the lower limbs
ZRS 406 A>G	Werner syndrome phenotype
ZRS 417 A>G	Mirror image polydactyly in all 4 limbs and bilateral tibial defects
ZRS 619 C>T	PPD-LPAD associations confined to the upper limbs. The lower limbs are normal
*(B) Duplications*	
Microduplications (<80 kb) reported by Lohan et al. [15]	Laurin-Sandrow syndrome phenotype
96,605 bp duplication from nt 156,240,230 to nt 156,336,835 at 7q36.3	Syndactyly type IV with concurrent tibial hypoplasia

Werner syndrome: Tibial Hypoplasia, Polysyndactyly Triphalangeal Thumb Syndrome or THPTTS, OMIM 188770; Laurin-Sandrow syndrome: mirror-image polydactyly of the hands and feet, absent tibiae, and duplication of the fibulae; OMIM 135750; Syndactyly type IV: complete syndactyly of all digits with polydactyly, OMIM 186200.
